# Are life-saving anticancer drugs reaching all patients? Patterns and discrepancies of trastuzumab use in the European Union and the USA

**DOI:** 10.1371/journal.pone.0172351

**Published:** 2017-03-14

**Authors:** Felipe Ades, Christelle Senterre, Dimitrios Zardavas, Evandro de Azambuja, Razvan Popescu, Martine Piccart

**Affiliations:** 1 Department of Medical Oncology, Institut Jules Bordet, Université Libre de Bruxelles, Brussels, Belgium; 2 Centro de Oncologia e Hematologia, Hospital Israelita Albert Einstein, São Paulo, Brazil; 3 Research Center of Epidemiology, Biostatistics and Clinical Research, School of Public Health, Université Libre de Bruxelles, Brussels, Belgium; 4 Breast International Group Headquarters (BIG-aisbl), Brussels, Belgium; 5 Department of Medical Oncology, Hirslanden Clinic Aarau, Aarau, Switzerland; Azienda Ospedaliera di Cremona, ITALY

## Abstract

**Background:**

The development of trastuzumab is considered to be one of the greatest improvements in breast cancer treatment in recent years. This study aims to evaluate changes in the uptake of trastuzumab over the last 12 years and to determine whether its use is proportional to patient needs in the European Union and the USA.

**Methods:**

Using national registry data, the number of new cases of HER2-positive breast cancer patients per year was estimated. The number of likely trastuzumab treatments per year was estimated using trastuzumab procurement data for each country.

**Results:**

Western Europe and the USA show increasing procurement level of trastuzumab over the years studied, reaching proportional of use of trastuzumab few years after its marketing authorization in the early 2000’s. After the approval in the adjuvant setting, in the year 2006, it was observed underuse of trastuzumab given the increase of the number of patients in need of treatment. Proportional use was shortly met after a couple of years. Few countries in Eastern Europe acquired the needed quantity of trastuzumab, with procurement levels starting to increase only after approval in the adjuvant setting in 2006.

**Conclusion:**

Significant differences in trastuzumab procurement are observed between Western Europe, the USA and Eastern Europe, with the latter geographic region acquiring insufficient amounts of the drug required to treat all patients in need.

## Introduction

Breast cancer is the most common cancer among women in the USA and in the European Union (EU) [1]. Its incidence is rising across Europe, while it has been stable during the last decade in the USA. Despite this, breast cancer mortality is declining in both the EU and the USA [2,3].

The reduction in breast cancer mortality observed in the EU, however, is not homogeneous: it is more marked in Western Europe than in Eastern Europe [4]. Western European countries allocate more resources to health care than Eastern European countries [5], and this is associated with better survival after a cancer diagnosis [6]. This association is more marked in breast cancer, possibly because of the availability of effective screening methods, treatments, and multidisciplinary management [6]. In the USA, reductions in breast cancer mortality are also heterogeneous, being observed more in white women than in black, Hispanic and Native American women [3]. These results are probably related to multiple factors, such as genetic background, life style, and also access to health care systems. The US health care system relies mostly on private insurance companies, and around 15% of the population is not covered by any insurance [7].

One major aspect in reducing mortality and improving longevity is the development and uptake of health innovation and lifesaving cancer drugs. The development of new drugs has been estimated to be responsible for around 40% of the increase of life expectancy in recent decades [8].

The development of anti-HER2 agents, in particular trastuzumab, is considered to be one of the greatest improvements in breast cancer treatment in recent years. Around 15% to 20% of breast cancers show amplification of the HER2 gene [9]. The breast cancer subtype defined by this feature is known to have a more aggressive biological behavior and worse clinical prognosis. Trastuzumab is a monoclonal antibody targeting the extracellular domain of the HER2 receptor that has dramatically improved the outcome of patients with HER2-positive disease in both the metastatic [10] and in the adjuvant settings [11–15].

Trastuzumab was one of the first targeted drugs to receive European Medicine Agency (EMA) and North-American Food and Drug Administration (FDA) health authority approval for human use. With more than 15 years in the clinic, it is considered to be a highly effective treatment with a favorable benefit/risk profile; however, it comes at a high acquisition cost [16].

Delays in trastuzumab reimbursement approval in the EU were shown to be correlated with worse breast cancer survival [17], reinforcing the notion that the fast uptake of lifesaving drugs is important in cancer management. This study aims to evaluate changes in the clinical use of trastuzumab over the last 12 years, and whether its use is proportional to the need for it in the EU and USA.

## Material and methods

The primary objective of this study was to evaluate the use of trastuzumab in relation to the need for this drug in the EU and in the USA. Trastuzumab was chosen as a proxy of the drug uptake process in this study because of its high efficacy but at the expense of a high acquisition cost.

### The estimation of possible treated patients

**Fig 1:**
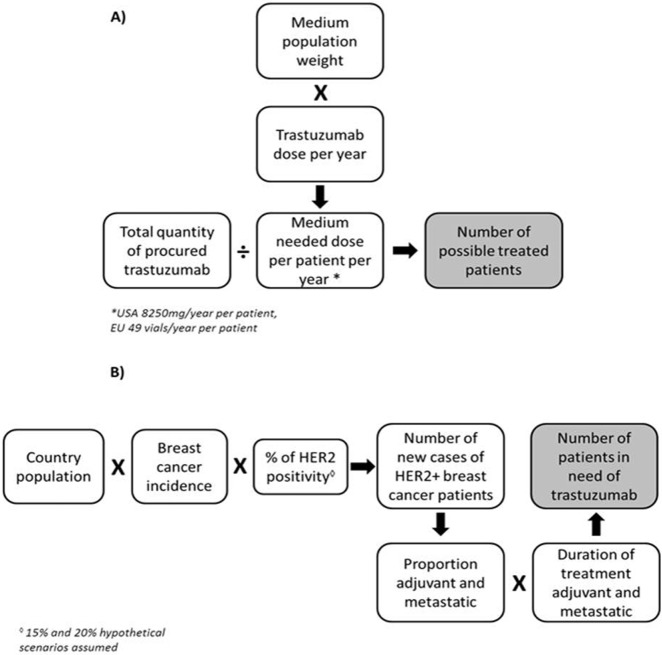
A) Estimation of the number of possible treated patients. B) Estimation of the number of patients in need of trastuzumab per year.

The number of possible patients treated with trastuzumab was estimated according to the number of procured vials, vial dose, and the median population weight (Fig 1A). Trastuzumab sales data in the EU and USA was acquired from IMS Health. Data from Cyprus, Estonia, Luxembourg and The Netherlands were not available, thus these countries were excluded from the analysis. Trastuzumab vials in Europe are presented in single dose formulations containing 150mg; in the USA they are presented in multidose formulations of 440mg.

According to the US national registry, the medium weight of North-American women is 74.7kg. The information regarding the medium weight of the female population in Europe is not available uniformly from the countries’ databases, it was then estimated according to results of previous peer-reviewed studies [18,19]. As these studies reported the medium weight of the total population, an evaluation of the national registries that discriminate weight per sex was carried out [20–22]. Weight gender differences ranged from 9.8kg to 15.2kg. It was assumed the female medium weight was similar across Europe and, on average, 5kg less than the medium total population weight. For the present analyses, the medium female weight in the USA was rounded to 75kg and in Europe to 65kg.

Based on these figures we estimated that the total dose per patient for one year of treatment in Europe would be 7150mg and in the USA 8250mg (8mg/kg loading dose + 6mg/kg for 17 cycles). In Europe the number of possible patients treated per year was calculated by dividing the number of acquired vials by 49, which is the number of 150mg single dose vials needed for one year of treatment. In the USA, because the vials are presented in 440mg multidose formulations, the number of possible patients treated was calculated by dividing the total number of milligrams of trastuzumab procured by 8250, the medium number of milligrams needed per patient per year.

### Estimation of patients needing trastuzumab

In order to calculate the number of patients needing trastuzumab per year, the following factors were considered: breast cancer incidence, the percentage of HER2-positive cases, the country population, the proportion of patients being treated in the metastatic and adjuvant settings, and metastatic treatment duration (Fig 1B).

To estimate the number of new cases of breast cancer per year and the proportion of patients treated in the adjuvant and metastatic settings, data from the World Health Organization (WHO) GLOBOCAN 2008 database was assessed. This year was chosen as being the nearest point to the medium year of the observation (from 2001 to 2013), when this information is available for all countries. Breast cancer indicators were considered to be stable across the years. The number of new cases of breast cancer per year was calculated taking into consideration the breast cancer incidence and the population size.

To calculate the number of HER2-positive cases, an evaluation of the national cancer registries was first carried out; however, HER2 data are not universally collected [23–25]. It was then decided to estimate the proportion of patients with HER2-positive disease based on data in the medical literature. No scientific evidence regarding differences in the incidence of HER2-positive breast cancer in Caucasian populations was found. According to the consolidated knowledge, HER2-positive breast cancer cases correspond from 15% to 20% of the new breast cancer cases [9,26]. The analyses were performed considering both scenarios.

The proportion of patients treated in the adjuvant and metastatic settings was calculated by dividing mortality by incidence. This value corresponds to the fraction of patients dying after a breast cancer diagnosis [27] and was taken to be the percentage of patients with metastatic breast cancer.

According to phase III studies in the HER2-positive metastatic setting, the medium treatment duration of a patient with anti-HER2 agents, can vary between 3.8 and 24.9 months depending on many factors such as previous treatment lines, chemotherapy combinations and disease subtypes [28–32]. Over the years, as knowledge on trastuzumab efficacy as second and third line treatment and its combinations with other agents was accumulated. It is likely that the “optimal” duration of treatment in the metastatic setting increased over time. We estimated then a figure for the median metastatic treatment duration, over the 12 years observed in this study, to be around 17 to 18 months.

### Over- versus underuse

Trastuzumab was approved in the metastatic setting in 1998 in the USA and in 2000 in Europe. An extended indication approval in the adjuvant setting was granted in 2006. For the years before 2006, only patients with metastatic breast cancer were considered to be “patients in need of treatment”. After publication of the results of studies of adjuvant trastuzumab treatment in late 2005, trastuzumab started being used in this setting in the USA (despite FDA approval only on 16 November 2006). This means that by 2006 in the USA, all new breast cancer patients were considered to be “in need of treatment”.

Because of the characteristics of the European health care systems, trastuzumab could not be administered in the adjuvant setting before EMA approval, which happened on 22 May 2006, around mid-year. For analysis purposes only, half of the expected patients in the adjuvant setting were considered to be “in need of treatment” in Europe in 2006. From 2007 onwards, all patients with HER2-positive breast cancer were viewed as requiring trastuzumab treatment.

The analysis to determine to which extent trastuzumab use is proportional to its need was made by dividing the number of possibly treated patients by the number of patients “in need of treatment” for each year. Values inferior to 1 represent underuse, and over 1, overuse.

### Relationship between trastuzumab landmarks and trastuzumab uptake

The dates of the publication of the results of the landmark phase III studies evaluating trastuzumab in breast cancer were extracted from the following studies: HERA [11], NSABP-B31 N9831 Joint Analysis [12], FinHER [33], PACS004 [14], BICRG006 [15]. The dates of trastuzumab marketing authorization approval in the metastatic and in the adjuvant breast cancer settings were extracted from the FDA [34] and EMA [35] websites. Most of the landmark studies were published around 2006, and the approval of trastuzumab in the adjuvant setting occurred in the same year. Analyses were performed to evaluate whether there were differences in trastuzumab uptake before and after this year.

### Statistical analysis

To ensure the comparison between the USA and the European countries, a coefficient equal to 2.93 was applied to American vials to compensate for the differences in vial dose (440mg vs 150mg). To compensate for the differences in the countries’ populations and breast cancer incidence, the ratio of vials per patient in need of treatment was used, taking in consideration both scenarios for HER2-positivity, namely 15% and 20%.

To estimate an average tendency across the years of the variation of vials and the over/underuse ratio, linear regressions were performed with the years as the independent variable. The Newey-West method was used to correct the standard error for the autocorrelation, which was verified by the Durbin-Watson test. Graphs were reported with the regression coefficients and 95% confidence intervals.

## Results

Given its much larger population than any in Europe, the USA is the country with the greatest absolute procurement of trastuzumab. The USA was also the first country to start using trastuzumab in the metastatic setting, with trastuzumab approved by the FDA two years before the EMA.

**Fig 2:**
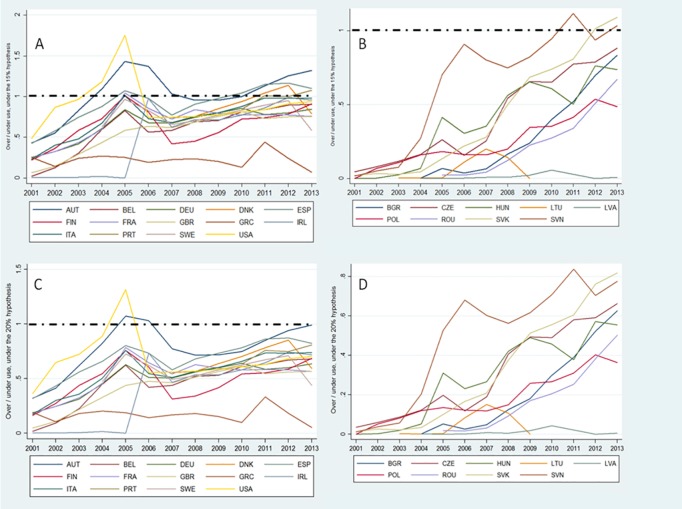
Over- versus underuse of trastuzumab. The vertical axis represents the percentage of possible patients treated with trastuzumab in relation to the number of patients in need of the drug. The value 1 represents proportional use of trastuzumab in relation to its need, values inferior to 1 indicates underuse and over 1 overuse. A) Western Europe and USA, 15% HER2+ scenario. B) Eastern Europe, 15% HER2+ scenario. C) Western Europe and USA, 20% HER2+ scenario. D) Eastern Europe, 20% HER2+ scenario.

The proportional use of trastuzumab in relation to its need was analyzed in two time periods. Before 2006, when trastuzumab was only approved in the metastatic setting, a sharp increase in use of trastuzumab was seen in the USA and in Western Europe, while in Eastern Europe small or no increases were seen. The USA was the first country to achieve use proportional to its demands. If we consider the 15% HER2-positive scenario, the USA reached complete coverage for patients with metastatic disease in 2003, while it did not happen for most of Western Europe until 2005. No Eastern European country procured trastuzumab in proportion to its needs (Fig 2a and 2b). When the 20% HER2 scenario is considered, only the USA and Austria reached a procurement level of trastuzumab proportional to their needs (Fig 2c and 2d).

After the publication of landmark phase III studies, trastuzumab became the “standard of care” in the adjuvant setting for HER2-positive breast cancer, receiving approval of both regulatory agencies in 2006. Because of this, the number of patients “in need” of trastuzumab increased dramatically in that year. The procurement of trastuzumab, however, did not increase by the same proportion. Considering the 15% HER2-positive scenario, the total (or quasi-total) coverage was achieved only several years later, between 2009 and 2013 in Western Europe and the USA.

Austria is an exception, indicating no underuse of trastuzumab since 2004, and appearing to have procurement even higher than its need. Greece is also an exception, showing a homogeneously low level of trastuzumab procurement across the years. There was a significant increase in the uptake of trastuzumab in Eastern Europe after 2006, but only Slovenia and the Czech Republic achieved the total procurement needed (Fig 2a and 2b). Considering the 20% HER2-positive scenario, all the figures are downgraded, with only Austria procuring enough trastuzumab for all patients (Fig 2c and 2d).

## Discussion

Trastuzumab is a monoclonal antibody that has changed the natural history of the HER2-positive breast cancer history, dramatically improving the outcome of these patients in the metastatic [36] and the in the adjuvant setting [11]. Given its outstanding activity in HER2 positive breast cancer patients and its high acquisition cost we hypothesized that trastuzumab uptake differences could be related to the discrepancies in breast cancer survival in the European Union.

Our results show significant difference in the trastuzumab uptake pattern between the EU countries and the USA. Historically the USA are the biggest buyer of cancer drugs, they represent 4.4% of the world population but procure 41% of all cancer drugs [37]. While most Western European countries and the USA procure the amount of trastuzumab necessary to treat virtually all patients in need of the drug, underuse of trastuzumab is observed in almost all Eastern European countries. The USA was the first country to approve trastuzumab in the metastatic setting, doing so around two years before the EU. The USA was also the first country to achieve a procurement level proportional to its need, this also happening around two years before Western Europe. The publication of the results of landmark adjuvant studies and the approval in the adjuvant setting in 2006 caused a significant change in trastuzumab uptake pattern in Eastern European countries, which at that time started or increased trastuzumab procurement.

The European population is passing though important demographic changes, it is expected, by the year 2050, that the population aged 65 years-old or more will rise from the nowadays 15% to 25% [38]. These changes will cause an important increase in the cancer incidence and mortality. Anticipating this future challenge the European Commission has created the Eurocadet project aiming to underpin European policies to prevent cancer. This effort has the purpose to estimate the potential impact of interventions directed to determinants of cancer incidence and the future burden of cancer in Europe [39]. Several research groups have also evaluated trends in cancer demographics in Europe [40–43]. Despite the increase in the new cases of cancer, in the recent update of the Eurocare-5 it was identified an increased survival in all European regions when comparing the year 2007 to the year 1999. Nevertheless survival was lower, and bellow the European median, in Eastern Europe “particularly for cancers with good or intermediate prognosis” [43].

The causes of the consistent increased cancer survival in Europe are likely to be multifactorial; Augmented health expenditure and better access to screening, specialized diagnostics and treatment [6,41–43]. Nevertheless controversy exists about the role of delayed diagnosis and access to new expensive cancer drugs in improving survival [44]. With the rising cost of cancer care such discussions are timely and necessary; for improving and providing affordable cancer care it is absolutely necessary to identify medical interventions that are clinically meaningful in real life and not only statistically significant in the research setting with a marginal clinical benefit.

Our study used models for estimating patients in need of trastuzumab and the number of possible treatments. The model had to be developed because it would be impossible to assess individual patient data, not only because of privacy issues but also because this data is not collected in such way or not collected at all. Models are used as a proxy of the subject of study and cause inherent limitations in the interpretation of the data, despite any control one might add to them. To create our model we had to make assumptions. Because of issues with data availability it was assumed that breast cancer incidence was stable during the 12 observed years. We also assumed that the median population weight was similar across the European countries. Breast cancer indicators are evaluated periodically by the World Health Organization (WHO) in the GLOBOCAN project; There were 3 versions available in the observation period of our study; from the years 2002, 2008 and 2012. The 2008 edition was chosen because this was the closest year to the medium point of the observations (from 2002 to 2013). For the estimation of the female weight, data from peer-reviewed studies was used because there is no standardized collection of this data in the national registries of the EU member states. The model also assumed the metastatic treatment time was stable during all the period. During the 12 years of the observation period trastuzumab was shown to be effective in second and third lines of treatment, also new drugs were developed and the combination of trastuzumab with these drugs have improved survival and time on treatment of breast cancer patients in the metastatic setting, as for example the combinations of trastuzumab with lapatinib [45], aromatase inhibitors [46] or pertuzumab [47]. According to peer review studies we chose a number in the middle of the observation for the metastatic treatment duration and the “expected metastatic treatment duration” was considered equal to all countries. In that way the imprecisions it generates could impact the absolute result of each country however they do not influence the comparative analysis. One interesting observation, in that regard, is the crescent procurement level of trastuzumab, beyond our estimate of proportional use in some countries. This may reflect the improvement of treatment in the metastatic setting, which is now longer and requires a larger quantity of the drug.

Despite of the limitations of the model, an observation that reassures its performance was that the procurement levels reach the proportional use the USA and several countries Western European countries. Marked overuse of trastuzumab was not seen in any country and, in countries that underused trastuzumab, worse breast cancer survival was observed, confirming the study hypothesis. The results were also are consonant with epidemiological data and previous peer-reviewed studies [4,6,48–50].

This is a case study analysis of a high-cost, life-saving drug that is used only by a fraction of the breast cancer patient population; being part of a larger process, it provides insight into the pattern of drug use and access, showing a large implementation variation of trastuzumab among countries with different patterns of health expenditure. Advances in all areas of health care, ranging from screening [51–53] to surgery and radiotherapy [54], endocrine treatment [55], and chemotherapy [56], have contributed to the decreasing breast cancer mortality trend in the USA and Europe. The demonstration of the higher trastuzumab uptake in countries with higher breast cancer survival strengthens the notion that the uptake of life-saving drugs is one, of many, important factors in improving breast cancer survival.
